# Maize MeJA-responsive proteins identified by high-resolution 2-DE PAGE

**DOI:** 10.1016/j.dib.2015.08.034

**Published:** 2015-09-07

**Authors:** Yuliang Zhang, Kayla K. Pennerman, Fengshan Yang, Guohua Yin

**Affiliations:** a Key Laboratory of Biology and Genetic Resources of Tropical Crops, Ministry of Agriculture, Institute of Tropical Bioscience and Biotechnology, Chinese Academy of Tropical Agricultural Sciences, Haikou, Hainan 571101, China; b Department of Plant Biology and Pathology, Rutgers, The State University of New Jersey, New Brunswick, New Jersey, United States; c Key Laboratory of Molecular Biology of Heilongjiang Province, College of Life Sciences, Heilongjiang University, Harbin 150080, China

## Abstract

Exogenous methyl jasmonate (MeJA) is well-known to induce plant defense mechanisms effective against a wide variety of insect and microbial pests. High-resolution 2-DE gel electrophoresis was used to discover changes in the leaf proteome of maize exposed to MeJA. We sequenced 62 MeJA-responsive proteins by tandem mass spectroscopy, and deposited the mass spectra and identities in the EMBL-EBI PRIDE repository under reference number PXD001793. An analysis and discussion of the identified proteins in relation to maize defense against Asian corn borer is published by Zhang et al. (2015) [1].

## 1 Specifications table

**Table:** 

Subject area	Biology, Plant Pathology
More specific subject area	Volatile responses, plant defense, proteomics, 2-DE gel images
Type of data	2-DE gel images and protein sequences from tandem mass spectroscopy and their putative identities
How data was acquired	2-DE gels were examined with a digital imager to find differentially-accumulated proteins that were sequenced by mass spectroscopy. Protein sequences were compared to others in public databases to assign putative identities and functions
Data format	Available in PRIDE Archive in table format
Experimental factors	Exogenous MeJA
Experimental features	MeJA was applied to experimental maize leaves before protein extraction and subjection to 2-DE gel electrophoresis. Differentially-accumulated proteins were detected and sequenced by tandem mass spectroscopy
Data source location	Harbin, Heilongjiang province, China and Gainesville, Florida, USA
Data accessibility	In this article and at EMBL-EBI PRIDE repository under reference number PXD001793: http://www.ebi.ac.uk/pride/archive/projects/PXD001793

## 2 Value of the data

• Proteins likely involved in maize MeJA response and signal transduction were identified.
• Future development of pest-resistant cultivars may target these proteins.
• The proteins may be compared with proteomic responses to biotic pests to identify responses not strictly from jasmonate responses.

## 3 Data, experimental design, materials and methods

Sixty-two maize proteins reproducibly differentially-accumulated in response to exogenous MeJA treatment were identified by 2-DE PAGE. These were subjected to mass spectroscopy and bioinformatics analysis for sequencing and identification.

### 3.1 Plant materials, growth and MeJA exposure

Seeds of *Zea mays* L. Dongnong 250 were sown into plastic basins (1×15 cm^2^) and kept at room temperature. The plants were grown at 27+1 °C with a 14:10 h (light:dark) photoperiod with 80% relative humidity. When the maize plants developed to the interior leaf period, they were treated with 225 µM of MeJA for 12 h, frozen in liquid nitrogen and stored at −80 °C for protein extraction. Three batches of biological replicates were collected for both control and treated samples.

### 3.2 Protein extraction

After MeJA treatment, corn leaves were grounded into powder in liquid nitrogen. Proteins were precipitated in a 10% TCA, cold acetone solution containing 0.07% β-mercaptoethanol at −20 °C for two hours. After centrifugation at 40,000*g* at 4 °C for one hour, the supernatant was discarded and the pellet was rinsed with −20 °C cold acetone containing 0.07% β-mercaptoethanol. The final pellet was vacuum-dried and solubilized on ice for about one hour in a 3 mL solution of 7 M urea, 2 M thiourea, 40 mM DTT and 1% protease inhibitor mixture (GE Healthcare, USA). Insoluble materials were removed by centrifugation at 100,000 rpm for one hour. The protein concentration was determined using the 2-DE Quant kit (GE Healthcare, USA) with BSA as a standard. Samples were frozen in liquid nitrogen and stored at −80 °C for further analysis.

### 3.3 2-DE gel electrophoresis

Proteins from the control group and MeJA-treated plants were compared using 2-DE gel image analysis. The isoelectric points of the spots ranged from 4 to 7, and the molecular masses ranged from 10 to 120 kDa. For each sample, 1 mg total protein in 450 µL rehydration buffer (7 M urea, 2 M thiourea, 2% CHAPS, 0.5% IPG buffer with PH 4–7, 0.04 M DTT) was loaded onto a 24 cm, pH 4–7 linear gradient IPG strip (GE Healthcare, USA). Isoelectric focusing was performed using an Ettan IPGphor 3 isoelectric focusing (IEF) system according to the manufacturer's instructions. The focusing conditions were as follows: active rehydration was carried out at low voltage for 12 h, followed by 300 V for 1 h, 600 V for 1 h, 1000 V for 1 h, with a linear increase of voltage to 8000 V for 12 h at 20 °C. The voltage was held at 10,000 V until the total voltage hours reached 80,000. After IEF, the strips were equilibrated with an equilibration solution (50 mM Tris with pH 8.8, 6 M urea, 30% glycerol, 1% DTT, 2% SDS) followed by alkylation in equilibration solution with 2.5% iodoacetamide, each for 15 min. The second dimension was performed on the 12.5% polyacrylamide gels using an Ettan DALT Six Electrophoresis Unit (GE Healthcare, USA) according to the manufacturer's instructions. The 2-DE gel electrophoresis experiments were repeated three times using protein samples prepared independently from MeJA treated and control maize.

### 3.4 Detection and identification of MeJA-responsive proteins

**Fig. 1:**
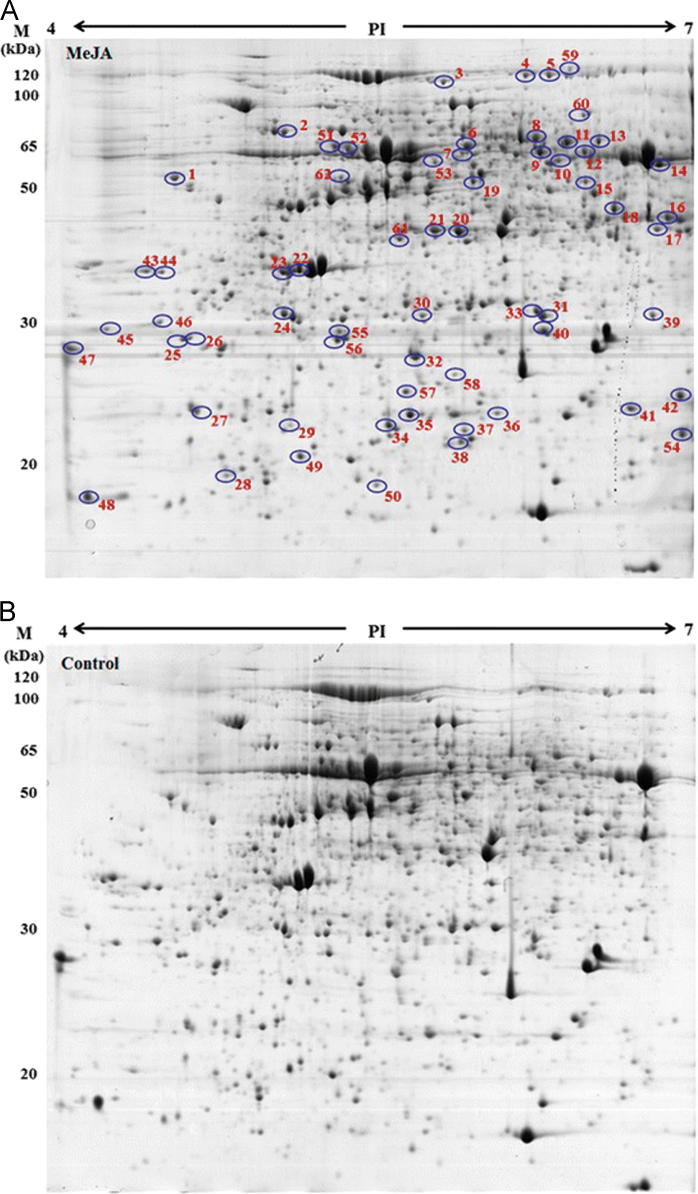
Comparison of representative 2-DE gel images of maize leaf proteome (A) after treatment with 225 μM MeJA for 12 h and (B) from control plants. 1 mg of protein was loaded on the IPG strips, and the proteins were visualized using Coomassie Brillant Blue. Differentially-accumulated proteins are circled and numbered. These images are also published by Zhang et al. [1].

Proteins were visualized with Coomassie Brilliant Blue R250 and gel images (Fig. 1) were acquired using an ImageScanner (GE Healthcare, USA). Replicate gels from control and MeJA treatment were analyzed with ImageMaster 2-D Platinum Software Version 7.0 (Amersham Biosciences, USA). The experimental molecular weight (kDa) of each protein was estimated by comparison with the protein markers, and experimental isoelectric points were determined by migration distance on the IPG strip. The abundance of each protein spot was estimated by the percent volume. Only those spots with significant and reproducible changes were considered to be differentially expressed proteins. The normalized volumes of the spots from replicate gels were subjected to student's ANOVA test (*p*<0.05) and only statistically significant data were considered. Protein in-gel tryptic digestion and nanoESI MS/MS analysis were carried out on a QSTAR XL MS/MS system (AB Sciex Inc., USA) as previously described [2]. The peptide MS/MS spectra were searched against an NCBI non-redundant fasta database using the Mascot search engine (http://www.matrixscience.com). Mascot was set up to search green plants only, assume trypsin digestion and allow one miscleavage. The mass tolerances for both parent ion and fragment ion masses were set to be 0.2 Da. Iodoacetamide derivatization of Cys, deamidation of Asn and Gln, and oxidation of Met were specified as variable modifications. We initially performed a fixed modification analysis and found that most of the differential protein spots had Cys modifications. Thus, we set iodoacetamide derivization of Cys as a variable modification to obtain better alignments. Unambiguous identification was judged by the number of peptides, sequence coverage, Mascot score and the quality of MS/MS spectra.

## Conflicts of interest

None.
